# Outcomes following extended thoracic endovascular aortic repair for Type B aortic dissection from the global registry for endovascular aortic treatment

**DOI:** 10.1093/icvts/ivaf156

**Published:** 2025-07-17

**Authors:** Sara Allievi, Tim J Mandigers, Fred A Weaver, Ali Azizzadeh, Gabriele Piffaretti, Marc L Schermerhorn, Gregory A Magee, Dennis R Gable, Chiara Lomazzi, Santi Trimarchi

**Affiliations:** Section of Vascular Surgery, Cardio Thoracic Vascular Department, Foundation IRCCS Ca’ Granda Ospedale Maggiore Policlinico, Milan, Italy; Division of Vascular and Endovascular Surgery, Department of Surgery, Beth Israel Deaconess Medical Center, Harvard Medical School, Boston, MA, USA; Section of Vascular Surgery, Cardio Thoracic Vascular Department, Foundation IRCCS Ca’ Granda Ospedale Maggiore Policlinico, Milan, Italy; Division of Vascular and Endovascular Surgery, Department of Surgery, Beth Israel Deaconess Medical Center, Harvard Medical School, Boston, MA, USA; Department of Vascular Surgery, University Medical Center Utrecht, Utrecht, The Netherlands; Division of Vascular Surgery and Endovascular Therapy, Department of Surgery, Keck School of Medicine, University of Southern California, Los Angeles, CA, USA; Division of Vascular Surgery, Cedars-Sinai Medical Center, Los Angeles, CA, USA; Vascular Surgery, Department of Medicine and Surgery, University of Insubria School of Medicine, Varese, Italy; Division of Vascular and Endovascular Surgery, Department of Surgery, Beth Israel Deaconess Medical Center, Harvard Medical School, Boston, MA, USA; Division of Vascular Surgery and Endovascular Therapy, Department of Surgery, Keck School of Medicine, University of Southern California, Los Angeles, CA, USA; Division of Vascular and Endovascular Surgery, Baylor Scott & White Heart Hospital, Plano, TX, USA; Section of Vascular Surgery, Cardio Thoracic Vascular Department, Foundation IRCCS Ca’ Granda Ospedale Maggiore Policlinico, Milan, Italy; Section of Vascular Surgery, Cardio Thoracic Vascular Department, Foundation IRCCS Ca’ Granda Ospedale Maggiore Policlinico, Milan, Italy; Department of Clinical Sciences and Community Health, University of Milan, Milan, Italy

**Keywords:** thoracic endovascular aortic repair, Type B aortic dissection, spinal cord ischaemia, sac dynamics, GREAT registry, long-term outcomes

## Abstract

**OBJECTIVES:**

This study evaluated the short- to longer-term safety and efficacy of extended thoracic endovascular aortic repair for Type B aortic dissection.

**METHODS:**

We identified acute and subacute Type B dissection between 2010 and 2016 in the Global Registry for Endovascular Aortic Treatment. We stratified the population based on treatment extent: ‘non-extended’ (1 stent graft deployed), ‘extended’ (>1 stent graft deployed). Our primary outcomes were in-hospital spinal cord ischaemia and mortality. Secondarily, we evaluated procedure-related complications (endoleak, migration, fracture, compression, reinterventions), sac dynamics, rupture, and all-cause mortality at 1 and 5 years. Sensitivity analysis was performed in patients without prior aortic procedures.

**RESULTS:**

Of 170 procedures, 78 (46%) were extended (median 2 [range 2–5] stent-grafts). Extended and non-extended treatment had similar rates of complicated presentations (53% vs 64%; *P* = 0.13). Compared with non-extended treatment, extended treatment had similar in-hospital rates of spinal cord ischaemia (2.6% vs 2.2%; *P* = 1) and mortality (2.6% vs 2.2%, *P* = 1). Additionally, extended treatment had not statistically different sac expansion rates, (1 year: 14% vs 23%, *P* =0.80; 5 years: 16% vs 32%, *P*=0.29) and rupture risk (1 year: 1.3% vs 3.3%, *P* = 0.63), similar procedure-related complications (endoleak, migration, fracture, compression, reinterventions; all *P* > 0.05) and all-cause mortality (1 year: 10% vs 7.6%, *P*=0.54; 5 years: 19% vs 21%, *P*=0.82). All outcomes remained similar on sensitivity analysis.

**CONCLUSIONS:**

Our findings suggest that extended treatment for Type B aortic dissection may be associated with similar procedure-related risks and complications. Future larger studies are needed to define who might benefit from an extended treatment and further optimize patient-specific treatment for aortic dissection.

## Graphical Abstract

**Figure:**
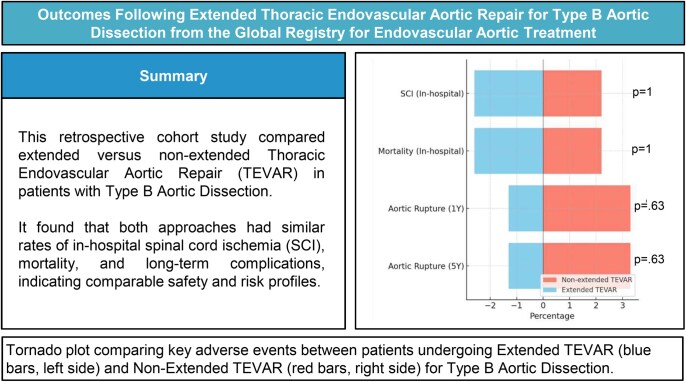

## INTRODUCTION

Type B aortic dissection (TBAD) poses significant cardiovascular threats, including aortic rupture, end-organ malperfusion, and aneurysmal degeneration. With the principal goal of covering the proximal entry tear to treat these complications, promoting false lumen (FL) thrombosis, and achieving favourable aortic remodelling, thoracic endovascular aortic repair (TEVAR) has been proposed as a treatment option [1–3] with comparable perioperative and mid-term results, both in complicated and uncomplicated TBAD [4].

However, lack of agreement remains regarding several important details of the procedure, especially the length of aortic coverage. Some authors already reported that extended TEVAR could improve FL remodelling in patients with TBAD, reducing the aortic disease and aortic-specific mortality [5, 6]. However, the need to cover all fenestrations between true lumen (TL) and FL in the thoracic aorta copes with a possible higher risk of postoperative complications, especially spinal cord ischaemia (SCI) [7]. Although the underlying mechanism of exclusion of direct intercostal perfusion appears intuitive, the occurrence of this complication is still variable and unpredictable. Prior studies already showed that length of aortic coverage may be an independent risk factor for SCI [8, 9]. However, most of the published studies are pertaining to thoracic aortic aneurysms, and data regarding aortic dissection are scarce [10].

The aim of this study was to analyse the safety and efficacy of extended aortic coverage in patients with TBAD. Understanding the risk–benefit ratio of extended TEVAR in the management of TBAD may offer crucial insights that could guide treatment decisions and potentially improve both short and long-term outcomes.

## METHODS

### Study design

This study is a retrospective observational cohort study analysing data obtained by the Global Registry for Endovascular Aortic Treatment (GREAT). The prospective, sponsored, multicentre, and observational GREAT database was designed to obtain real-world data on the performance of W.L. Gore & Associates (Flagstaff, AZ) endovascular aortic products and clinical outcomes of patients treated with these products (Clinicaltrials.gov identifier number: NCT01658787). The objectives and design, containing the precise inclusion and exclusion criteria of the registry, have been reported previously [11]. Enrollment started in December 2010 and was finalized in October 2016 with a target follow-up duration of 10 years. Each local participating centre must have obtained Institutional Review Board or Ethics Committee approval prior to participation, and written informed consent was obtained from all participants.

### Patient selection

The GREAT database was queried to obtain the data of patients with an acute or subacute TBAD who underwent TEVAR [4, 12, 13]. After excluding patients presenting with rupture, the overall cohort was stratified based on treatment extent (‘non-extended’ and ‘extended’).

**Figure 1:**
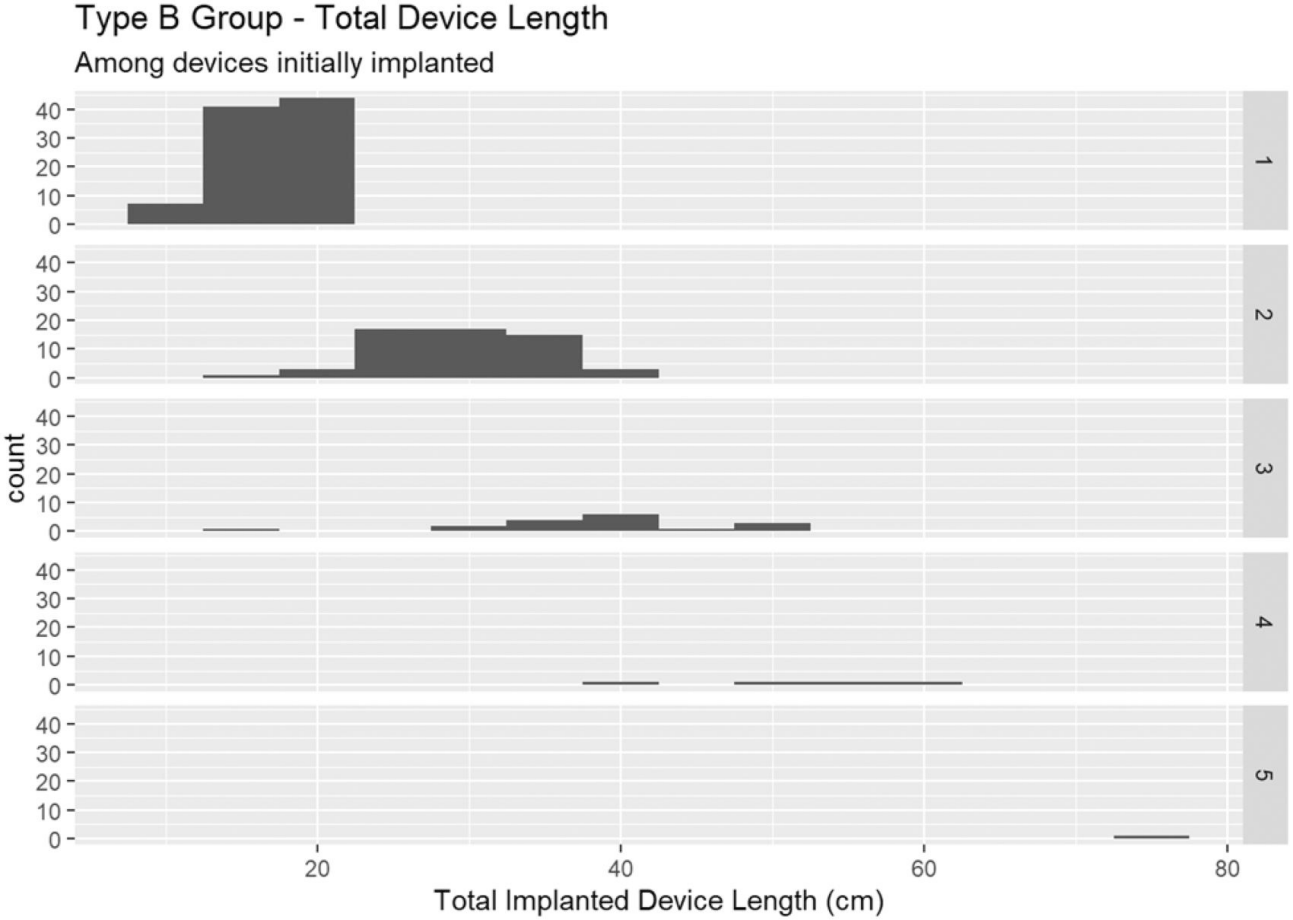
Number of patients undergoing TEVAR for Type B aortic dissection, categorized by length of aortic coverage and number of deployed stent-grafts

Extended aortic coverage was defined as >200 mm, in accordance with the literature [1]. Given the absence of specific variables for total aortic coverage, proximal and distal landing zone, and overlap between stent-grafts, ‘non-extended’ TEVAR was defined as 1 stent graft deployed, and ‘extended’ TEVAR was defined as >1 stent graft deployed. Only five subjects had ≤200 mm total device length after the deployment of >1 device (Fig. 1). Moreover, in the non-extended group, the vast majority of patients had <200 mm total device length. Therefore, even if the stent graft overlap was unknown, we believe treatment extent was adequately expressed by the number of devices.

### Outcomes definitions

The primary outcomes were in-hospital SCI and mortality. SCI was defined as any condition where blood flow to the spinal cord was compromised, leading to varying degrees of motor impairment. Secondary outcomes included postoperative complications at 1-year and 5-year follow-up. Outcomes included all-cause mortality, sac dynamics, endoleak, migration, fracture, compression, aortic rupture, and reinterventions [14]. Migration was defined according to the reporting standards as a stent graft shift of >10 mm relative to a primary anatomic landmark or any displacement that led to symptoms or required therapy. Stent graft compression was defined as concentric reduction of stent graft diameter within the proximal neck compared with the immediately adjacent portion of the stent graft. Sac expansion was defined as an increase of at least 5 mm, sac regression as a decrease of at least 5 mm, and stable sac size as a change <5 mm in either direction [15]. Baseline sac diameter was determined from imaging between days 1 and 59, and subjects without imaging in this window are excluded from sac expansion analysis.

### Statistical analysis

Continuous variables were presented as mean and standard deviation or median and interquartile range (IQR) where appropriate, based on the normal or non-normal distribution of the data. Categorical variables were presented as number and percentage. Comparisons between the non-extended TEVAR and extended TEVAR cohorts were performed with Fisher’s exact test, Wilcoxon rank sum test, or Pearson’s chi-squared test where appropriate.

Because prior aortic procedures might have compromised collateral circulation to the spinal cord [16], we performed a sensitivity analysis by restricting our population to the patients without prior aortic repair.

Lastly, potentially statistically significant monotonic upwards or downwards trends in the number of extended treatments over time were examined with the Mann–Kendall test.

Two-sided *P*-values <0.05 were considered statistically significant. All statistical analyses were performed using R version 4.3.3 (http://www.r-project.org).

## RESULTS

### Patients and procedural details

**Figure 2:**
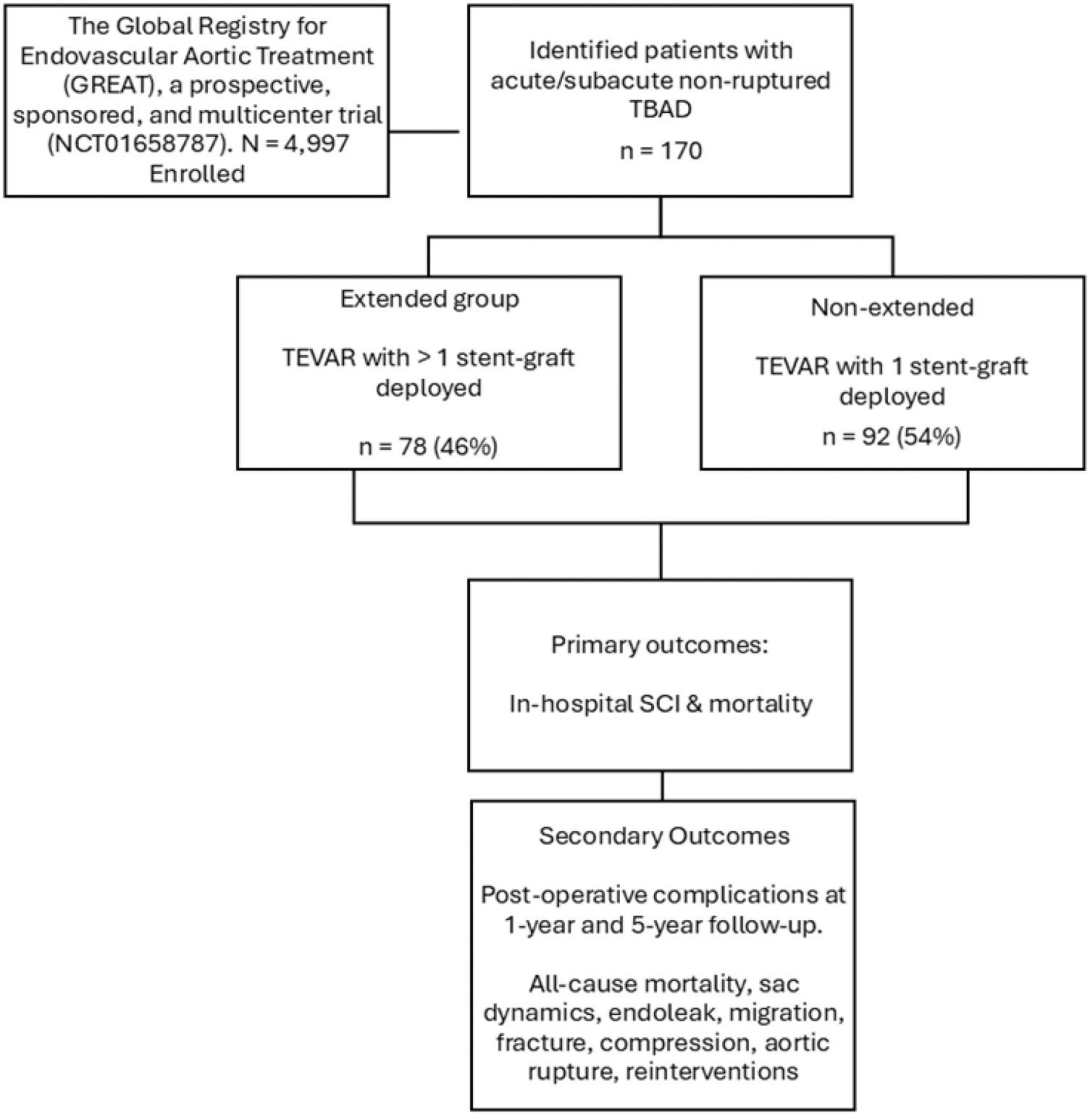
Patient flowchart detailing inclusion and exclusion criteria for study cohort

We identified 170 patients with acute and subacute non-ruptured TBAD, of whom 92 (54%) underwent non-extended TEVAR and 78 (46%) extended TEVAR (Fig. 2).

Between cohorts (Supplementary Material, Table S1), the comorbidity profiles were similar; however, compared with non-extended, extended TEVAR patients more frequently had valvular heart disease (17% vs 6.7%, *P*=0.041) and connective tissue disorders (CTD, 9.1% vs 0.0%, *P*=0.004), and less frequently had hypercholesterolaemia (26% vs 42%, *P*=0.035).

Compared with non-extended TEVAR (Supplementary Material, Table S2), extended TEVAR was equally performed for complicated TBAD (53% vs 64%, *P*=0.13), concomitant pathologies (descending/abdominal/thoraco-abdominal aneurysm, intramural haematoma, penetrating aortic ulcer; all *P* > 0.50), and as a revision of a prior stent graft (6.4% vs 7.6%, *P* =0.76). Extended TEVAR more frequently involved a combination of Gore TAG and Excluder stent-grafts (5.1% vs 0.0%, *P*=0.009). Extended TEVAR had a median of 2 deployed stent-grafts (IQR 2–3) with a mean length of 33 (9.6) cm (range 15–73 cm). Non-extended TEVAR had a mean length of 17 (3.2) cm (range 10–20 cm) (Fig. 1).

### Outcomes

Compared with non-extended TEVAR, extended TEVAR had similar in-hospital rates of SCI (2.6% vs 2.2%, *P* = 1) and mortality (2.6% vs 2.2%, *P* = 1). The two cohorts had comparable 1- and 5-year rates (Table 1) of procedure-related complications (endoleak, migration, fracture, compression, reinterventions; all *P*>0.05), all-cause mortality (1 year: 10% vs 7.6%, *P*=0.54; 5 years: 19% vs 21%, *P*=0.82), and aortic rupture (1 and 5 years: 1.3% vs 3.3%, *P*=0.63). Among subjects with baseline imaging, the cohorts had similar sac expansion rates (1 year: 14% vs 23%, *P*=0.80; 5 years: 16% vs 32%, *P*=0.29) and the groups had similar levels of missing baseline imaging data (extended: 39 [53%], non-extended 58 [60%]; χ^2^ = 0.45, *P*=0.50).

**Table 1:** Outcomes following TEVAR for Type B aortic dissection, stratified by treatment extent

	1 year					5 years				
	Non-extended		Extended		*P*-value	Non-extended		Extended		*P*-value
Endoleak	7	7.6%	5	6.4%	0.76	10	11%	8	10%	0.90
Type IA	2	2.2%	1	1.3%	1	4	4.3%	2	2.6%	0.69
Type IB	2	2.2%	1	1.3%	1	2	2.2%	1	1.3%	1
Type II	3	3.3%	0	0.0%	0.25	3	3.3%	1	1.3%	0.63
Type III	0	0.0%	1	1.3%	0.46	1	1.1%	1	1.3%	1
Type IV	0	0.0%	0	0.0%	.	0	0.0%	0	0.0%	
Migration	0.0%	0.0%	0.0%	0.0%	.	0	0.0%	0	0.0%	
Fracture	0.0%	0.0%	0.0%	0.0%	.	0	0.0%	0	0.0%	
Compression	0.0%	0.0%	0.0%	0.0%	.	0	0.0%	0	0.0%	
Reinterventions	15	16%	13	17%	0.95	21	23%	14	18%	0.43
Open conversion	1	1.1%	3	3.8%	0.33	1	1.1%	4	5.1%	0.18
Additional graft	3	3.3%	2	2.6%	1	6	6.5%	3	3.8%	0.51
Other	12	13%	10	13%	0.97	16	17%	11	14%	0.56
Device related	10	11%	10	13%	0.69	14	15%	11	14%	0.84
Aortic rupture	3	3.3%	1	1.3%	0.63	3	3.3%	1	1.3%	0.63
Aortic-related mortality	2	2.2%	3	3.8%	0.66	2	2.2%	3	3.8%	0.66
All-cause mortality	7	7.6%	8	10%	0.54	19	21%	15	19%	0.82
Sac dynamics^a^					0.80					0.29
Regression	5/22	23%	7/28	25%		6/25	24%	12/32	38%	
Stable	12/22	55%	17/28	61%		11/25	44%	15/32	47%	
Expansion	5/22	23%	4/28	14%		8/25	32%	5/32	16%	

a Among subjects that have any post-procedure imaging and available in time window.

### Sensitivity analysis: outcomes in patients without prior aortic repair

Of 170 patients, 130 (76%) did not have prior aortic repair, of whom 59 (45%) underwent extended TEVAR (median 2 [range 2–4] stent-grafts). Outcomes remained similar compared with the full population: in particular, compared with non-extended TEVAR, extended TEVAR had similar in-hospital rates of SCI (2.6% vs 2.2%, *P*>0.90) and mortality (2.6% vs 2.2%, *P*>0.90). Extended and non-extended TEVAR had comparable 1- and 5-year rates (Table 2) of procedure-related complications (endoleak, migration, fracture, compression, reinterventions; all *P*>0.05), and all-cause mortality (1 year: 10.3% vs 7.6%, *P* = 0.544; 5 years: 19.2% vs 20.7%, *P*=0.817). Moreover, extended TEVAR had similar risk of aortic rupture (1 and 5 years: 1.3% vs 3.3%, *P*=0.63) and sac expansion (1 year: 14.3%% vs 22.7%, *P* = 0.796; 5 years: 15.6% vs 32.0%, *P* = 0.289).

**Table 2:** Sensitivity analysis: outcomes following TEVAR for Type B aortic dissection, stratified by treatment extent, after excluding patients with prior aortic repair

	1 year					5 years				
	Non-extended		Extended		*P*-value	Non-extended		Extended		*P*-value
Endoleak	3	4.2%	4	6.8%	0.70	6	8.5%	7	12%	0.52
Type IA	1	1.4%	1	1.7%	1	3	4.2%	2	3.4%	1
Type IB	0	0.0%	1	1.7%	0.45	0	0.0%	1	1.7%	0.45
Type II	1	1.4%	0	0.0%	1	1	1.4%	1	1.7%	1
Type III	0	0.0%	0	0.0%	.	0	0.0%	0	0.0%	.
Type IV	0	0.0%	0	0.0%	.	0	0.0%	0	0.0%	.
Migration	0	0.0%	0	0.0%	.	0	0.0%	0	0.0%	.
Fracture	0	0.0%	0	0.0%	.	0	0.0%	0	0.0%	.
Compression	0	0.0%	0	0.0%	.	0	0.0%	0	0.0%	.
Reinterventions	10	14%	10	17%	0.65	15	21%	11	19%	0.73
Open conversion	1	1.4%	2	3.4%	0.59	1	1.4%	3	5.1%	0.33
Additional graft	2	2.8%	2	3.4%	1	3	4.2%	3	5.1%	1
Other	8	11%	8	14%	0.69	12	17%	9	15%	0.80
Device related	6	8.5%	7	12%	0.52	10	14%	8	14%	0.93
Aortic rupture	3	4.2%	1	1.7%	0.63	3	4.2%	1	1.7%	0.63
Aortic-related mortality	2	2.8%	2	3.4%	1	2	2.8%	2	3.4%	1
All-cause mortality	7	9.9%	5	8.5%	0.79	16	22%	11	19%	0.59
Sac dynamics^a^					0.51					0.84
Regression	5/15	33%	3/18	17%		5/16	31%	6/21	29%	
Stable	7/15	47%	12/18	67%		6/16	38%	10/21	48%	
Expansion	3/15	20%	3/18	17%		5/16	31%	5/21	24%	

a Among subjects that have any post-procedure imaging and available in time window.

### Trend analysis

**Figure 3:**
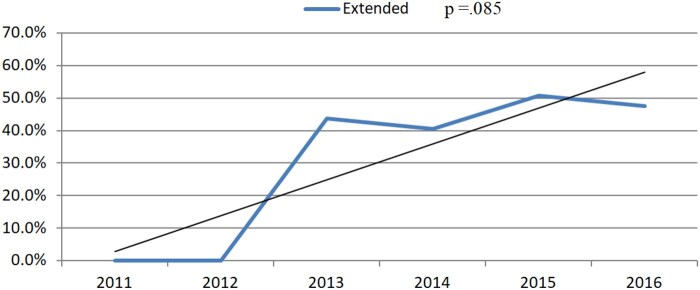
Proportion of patients receiving extended TEVAR for Type B aortic dissection over the study period

We observed an increase in the application of extended TEVAR (2011–2016: 0–47%, *P*=0.085) and a decrease in the application of non-extended TEVAR over the study period (2011–16: 100–53%, *P* =0.085); however, this was not statistically significant on trend analysis (Fig. 3).

## DISCUSSION

The treatment of TBAD is hindered by a lack of agreement, particularly regarding the optimal length of aortic coverage. While supporters of extended TEVAR advocate for improved aortic remodelling and long-term outcomes, others argues that this strategy might increase the risk of SCI. Therefore, we compared short- to long-term outcomes of extended and non-extended TEVAR for acute and subacute TBAD. Data suggested that patients undergoing extended TEVAR had similar risk of in-hospital SCI and mortality, procedure-related complications, and all-cause mortality at 1 and 5 years.

The aim of TEVAR for treating complicated TBAD or uncomplicated TBAD with high-risk features is generally to cover the primary entry tear, promote FL thrombosis, and re-expand the TL. Prior studies reported length of aortic coverage as an independent risk factors for SCI, especially above 200 mm [8, 9, 17]. However, these studies are not only pertaining to aortic dissection and the number of events is too low to draw any solid conclusion. In our population, the rate of SCI was low (2.3%) and comparable to those reported in the literature (1.3%) [18]. Moreover, we found that compared with non-extended TEVAR, extended TEVAR was associated with similar risk of in-hospital SCI. The association between prior AAA repair and SCI has also been inconsistent among published reports. The results in our study were similar to the results of the TAG pivotal trial, which did not find any difference in the rates of SCI between those with and without prior AAA repair following TEVAR for thoracic aneurysms [19]. A history of abdominal aortic replacement is likely a surrogate marker for decreased spinal cord perfusion as it relates to the lumbar arteries. Like the anterior spinal artery, the contribution of this or any other single blood supply to the development of clinically significant SCI cannot be easily determined [20].

A patent or partially thrombosed FL after TEVAR is a predictor of complications after TBAD, since a persistent pressurization of the FL can lead to aortic-related events. A recent systematic review evaluated 16 studies and concluded that a complete FL thrombosis was associated with smaller yearly aortic growth rate and thus a lower risk of aortic rupture and hospital death [21]. Prior studies reported that extended TEVAR could improve FL remodelling in patients with TBAD by covering distal entry tears and avoiding residual aortic dissection [5, 6]. However, these studies suffered small sample sizes, different definitions of extended treatment, and inclusion/exclusion criteria. Our study adds to the existing literature, since GREAT is one of the largest worldwide registries with granular data to study our specific outcomes in TBAD. Moreover, extended TEVAR had similar procedure-related short- to long-term risks and complications. Unfortunately, the sample size did not allow us to draw any solid conclusions on the differences in sac dynamics and rupture risk.

Several techniques have been proposed to promote FL thrombosis [22–27], and these methods can be employed alongside both extended and non-extended TEVAR. Further studies with longer follow-up and larger sample size will help define a patient-specific treatment for TBAD and improve overall outcomes. Moreover, the role of computational fluid dynamics analysis is emerging [28–30] and might help in implementing a tailored risk prediction model in TEVAR for TBAD.

Extended TEVAR patients more frequently had CTD. The development of new technologies and devices has broadened the application of endovascular interventions [31]; however, due to concerns of vessel fragility, risk of retrograde dissections, and durability, endovascular repair in patients with CTD is reserved for selected cases [2, 32, 33]. Additionally, as CTD was an exclusion criterion for pivotal trials leading to all FDA-approved stent-grafts, controversy remains regarding the benefits and risks of stent graft deployment in CTD patients [34]. However, TEVAR may be the primary life-saving option in case of rupture, complex dissection anatomy, and malperfusion, which appeared to be more common in these patients [34]. Many CTD patients will require extensive treatment with aortic branch stenting in addition to aortic repair or TEVAR to solve malperfusion upon presentation. Initiatives like the Endovascular Aortic Intervention in Patients With Connective Tissue Disease (EVICTUS) registry will yield newer perspectives and help defining the best treatment for this group of high complexity patients [35, 36].

We believe that each case should be examined individually, keeping in mind the importance of spinal cord protection (avoid intraoperative prolonged hypotension, left subclavian artery revascularization whenever feasible, cerebral fluid drainage in high-risk patients) as recommended by the guidelines [1–3].

### Limitations

The results of this study must be interpreted in the context of its design. First, the small sample size and the low number of events could have had an impact on detecting possible differences between the two cohorts. However, GREAT is one of the largest worldwide registries with granular data to study our specific outcomes in TBAD. The lack of imaging and data on the actual length of aortic coverage and device overlap, proximal and distal seal zones, and adjunctive procedures for FL thrombosis must also be acknowledged. Given the definition of extensive aortic coverage by the European Society for Vascular Surgery, however [1], the number of deployed stent graft was deemed an adequate surrogate of treatment extent. The absence of variables for spinal cord protection or treatment strategies, intraoperative pressure, hypogastric arteries patency, FL thrombosis, and disease extension might have also had an impact on the conclusions.

## CONCLUSIONS

Our findings suggest that extended TEVAR for TBAD might be associated with similar procedure-related short- to long-term risks and complications. Future larger studies with more granular data are needed to define who might benefit from an extended treatment and to further optimize patient-specific treatment for TBAD.

## Supplementary Material

ivaf156_Supplementary_Data

## Data Availability

The data underlying this article were provided by W.L. Gore and Associates by permission. Data will be shared on request to the corresponding author with permission of W.L. Gore and Associates.

## ABBREVIATIONS

CTD: Connective tissue diseases
FL: False lumen
GREAT: Global Registry for Endovascular Aortic Treatment
IQR: Interquartile range
SCI: Spinal cord ischaemia
SVS: Society for Vascular Surgery
TBAD: Type B aortic dissection
TEVAR: Thoracic endovascular aortic repair
TL: True lumen
