# Supplementary material for: Outcomes following extended thoracic endovascular aortic repair for Type B aortic dissection from the global registry for endovascular aortic treatment
Source: Interdiscip Cardiovasc Thorac Surg. 2025 Jul 17;40(7):ivaf156. doi: 10.1093/icvts/ivaf156 (PMC12282756; doi:10.1093/icvts/ivaf156)
Supplement: ivaf156_Supplementary_Data [file ivaf156_supplementary_data.docx]

Table S1. Sensitivity analysis: demographics and comorbidities of patients undergoing TEVAR for type B aortic dissection (only patients without prior aortic repair).

|  | **Non-Extended**  **N = 71** | | **Extended**  **N = 59** | | **p-value** |
| --- | --- | --- | --- | --- | --- |
| **Age, years** | 63 | [54, 70] | 63 | [58, 71] | .62 |
| **Female Gender** | 15 | 21% | 16 | 27% | .43 |
| **Race** |  |  |  |  | .36 |
| *White* | 45 | 63% | 41 | 70% |  |
| *Black* | 12 | 17% | 13 | 22% |  |
| *Other* | 7 | 9.9% | 4 | 6.8% |  |
| *Unknown* | 7 | 9.9% | 1 | 1.7% |  |
| **BMI** | 27 | [25, 32] | 28 | [25, 31] | .87 |
| **Tobacco Use** | 33 | 50% | 28 | 52% | .84 |
| **Hypercholesterolemia** | 28 | 41% | 12 | 21% | **.019** |
| **Hypertension** | 63 | 89% | 52 | 88% | .92 |
| **Diabetes Mellitus** | 9 | 13% | 0 | 0.0% | **.004** |
| **Coronary Artery Disease** | 12 | 17% | 6 | 10% | .26 |
| **Cardiac Arrhythmia** | 13 | 18% | 5 | 8.5% | .11 |
| **Valvular Heart Disease** | 5 | 7.1% | 6 | 10% | .54 |
| **Congestive Heart Failure** | 4 | 5.7% | 5 | 8.5% | .73 |
| **Coronary Artery Bypass** | 0 | 0.0% | 1 | 1.7% | .45 |
| **COPD** | 7 | 9.9% | 7 | 12% | .71 |
| **Renal Insufficiency** | 14 | 20% | 11 | 19% | .88 |
| **Dialysis** | 1 | 1.4% | 1 | 1.7% | 1 |
| **Transient Ischemic Attack** | 1 | 1.4% | 1 | 1.7% | 1 |
| **Stroke** | 4 | 5.7% | 1 | 1.7% | .37 |
| **Peripheral Vascular Disease** | 4 | 5.7% | 6 | 10% | .51 |
| **Cancer** | 3 | 4.3% | 5 | 8.6% | .47 |
| **Connective Tissue Disorders** | 0 | 0.0% | 3 | 5.1% | .10 |
| **Paraplegia** | 1 | 1.4% | 1 | 1.8% | 1 |
| **Paraparesis** | 1 | 1.4% | 0 | 0.0% | 1 |

Table S2. Sensitivity analysis: pathology and procedural details of patients undergoing TEVAR for type B aortic dissection, stratified by treatment extent (only patients without prior aortic repair).

|  | **Non-Extended**  **N = 71** | | **Extended**  **N = 59** | | **p-value** |
| --- | --- | --- | --- | --- | --- |
| **Indication for Repair** |  |  |  |  |  |
| *Type B Complicated* | 48 | 68% | 34 | 58% | .24 |
| *Type B Uncomplicated* | 23 | 32% | 25 | 42% | .24 |
| *Descending Thoracic Aneurysm* | 2 | 2.8% | 0 | 0.0% | .50 |
| *Arch Aneurysm* | 0 | 0.0% | 1 | 1.7% | .45 |
| *Thoracoabdominal Aneurysm* | 0 | 0.0% | 1 | 1.7% | .45 |
| **Revision of Prior Stent-graft** | - | - | - | - | - |
| **Device Type** |  |  |  |  | **.047** |
| *Conformable TAG* | 63 | 89% | 54 | 92% |  |
| *TAG* | 8 | 11% | 2 | 3.4% |  |
| *Other Combination* | 0 | 0.0% | 3 | 5.1% |  |
| **Off Label** | 32 | 45% | 26 | 44% | .91 |
| **Off Indication** | 9 | 13% | 6 | 10% | .66 |
| **Off Direction** | 23 | 32% | 20 | 34% | .86 |
| **Incorrect Proximal Diameter** | 7 | 9.9% | 4 | 6.8% | .75 |
| **Incorrect Distal Diameter** | 16 | 22% | 9 | 15% | .29 |
| **Insufficient Landing Zone** | 17 | 24% | 18 | 31% | .40 |
| **Chimney Procedure** | 1 | 1.4% | 0 | 0.0% | 1 |
| **Improper Placement** | 1 | 1.4% | 1 | 1.7% | 1 |
| **LSA Procedure** |  |  |  |  | .11 |
| *Chimney* | 1 | 1.4% | 0 | 0% |  |
| *Covered +/- embolization* | 12 | 17% | 6 | 10% |  |
| *Stent Only* | 2 | 2.8% | 4 | 6.8% |  |
| *Surgical de-branching* | 10 | 14% | 4 | 6.8% |  |
| *None* | 43 | 61% | 39 | 66% |  |
| *Stent only and Surgical de-branch* | 0 | 0% | 1 | 1.7% |  |
| *C +/- E &stent &surgical* | 1 | 1.4% | 0 | 0% |  |
| *C +/- E &surgical* | 2 | 2.8% | 5 | 8.5% |  |
